# HAPPY WHERE I AM: HOME AND COMMUNITY PREFERENCES, TELEHEALTH, SOCIAL ISOLATION, AND WELLNESS OF OLDER RURAL RESIDENTS

**DOI:** 10.1093/geroni/igz038.1296

**Published:** 2019-11-08

**Authors:** Cassandra Cantave Burton

**Affiliations:** AARP, Washington, D.C, United States

## Abstract

About 16 percent of adults 50-plus and 25 percent of 65-plus adults reside in rural areas or small towns in the United States . The percentage increases to rural communities could mean a higher prevalence of chronic disease, a higher disability rate, a lower prevalence of healthy behaviors, and a widening gap in life expectancy relative to the nation as a whole. Moreover, rural areas face additional obstacles and challenges such: Difficulty forming community partnerships because of proximity challenges; migration of younger individuals to cities for career and social opportunities, resulting in a smaller pool of potential caregivers; an aging housing stock that also may be unsafe and in need of repair; and inadequate resources available to meet the broad range of needs among older adults. AARP has been engaged with policy makers and community members to ensure that older residents who live in rural areas have access to community supports so they can remain in their homes and communities and have the services that they need as they get older. Presenters in this symposium will present data supporting AARP’s work to better the lives of older rural residents. Findings from AARP studies on home and community preferences, social isolation, telehealth and broadband access, and brain health will be presented.

